# Omega 3 fatty acid alleviates sleep deprivation‐induced oxidative stress, inflammation, and cognitive impairment in the hippocampus of Male Wistar rats

**DOI:** 10.1002/alz.088894

**Published:** 2025-01-03

**Authors:** Joy Ochai, Uduak Emmanuel Umana, Sunday Abraham Musa, Samuel B Oladele

**Affiliations:** ^1^ Federal University of Health Sciences Otukpo, Otukpo, Benue Nigeria; ^2^ Ahmadu Bello University Zaria, Zaria, kaduna Nigeria; ^3^ Ahmadu Bello University Zaria, Zaria, Kaduna Nigeria; ^4^ Ahmadu Bello University, Zaria, Kaduna Nigeria

## Abstract

**Background:**

Sleep deprivation leads to an increase in oxidative stress and activation of inflammatory response and both could increase the production and accumulation of toxic beta‐amyloid in the hippocampus which is considered one of the molecular drivers of Alzheimer’s pathogenesis and progression. Despite these findings, obtaining sleep is still challenging in our modern society that values work around the clock. Omega‐3 fatty acids represents an active biological agent with vital antioxidant and anti‐inflammatory activities that could protect memory in the face of insufficient sleep. This study evaluated the anti‐oxidative, anti‐inflammatory, and neurocognitive effect of omega‐3 fatty acids on memory impairment caused by sleep deprivation in male Wistar rats.

**Method:**

Thirty male Wistar rats were randomly divided into five groups of six rats each. Group I received 2ml/kg distilled water, Group II, III IV and V were sleep deprived for 18 hours daily for a 21 days duration using the modified multiple platform method. Additionally, group III, IV and V received 100mg/kg, 200mg/kg and 400mg/kg of omega‐3 fatty acids respectively via oral route. Neurobehavioral assay for spatial learning and memory using the Morris water maze (MWM) test was carried out. The animals were euthanised and the brain tissues excised, fixed, and processed for β‐amyloid study using Congo red stain. Tissue homogenate was used for assessment of malondialdehyde (MDA), superoxide dismutase (SOD), interleukin‐6 (IL‐6) and tumor necrosis factor‐alpha (TNF‐α) levels.

**Result:**

The result revealed a marked increase in the time‐spent in locating the escape platform in the MWM test, and an increased in β‐amyloid burden in the sleep deprived only group. Biochemical assessment also showed significant increase in MDA, IL‐6 and TNF‐ α and decrease in SOD level within this group. However, significant improvement in memory retention ability, decreased β‐amyloid accumulation, MDA, IL‐6, TNF‐ α and increase in SOD level were observed in the omega‐3 fatty acid treated groups. The observed changes were dose dependent.

**Conclusion:**

Omega‐3 fatty acids protects the hippocampus against memory impairment caused by sleep deprivation.

